# Real‐world clinical outcomes of olaparib therapy in Chinese patients with advanced serous ovarian cancer treated in Macau

**DOI:** 10.1002/cnr2.1180

**Published:** 2019-05-09

**Authors:** Yabing Cao, Hongtao Chen, Yaobin Huang, Hao Hu

**Affiliations:** ^1^ Department of Oncology Kiang Wu Hospital Macau SAR China; ^2^ Department of Laboratory the Fifth Affiliated Hospital of Sun Yat‐Sen University Zhuhai China

**Keywords:** BRCA mutation, China, Macau, olaparib, ovarian cancer

## Abstract

**Background:**

Olaparib has been approved as an active and maintenance therapy for patients with platinum‐sensitive, BRCA‐mutated high‐grade serous ovarian cancer (SOC). However, the efficacy and safety data is lack among Chinese ovarian cancer patients.

**Aim:**

This real‐world study aimed to evaluate the effectiveness and safety profile of olaparib in patients from mainland China, where olaparib is currently unavailable.

**Methods and results:**

This single‐center, observational study included 65 patients with pathologically confirmed advanced serous ovarian cancer from Kiang Wu Hospital in Macau between December 2015 and September 2017. Progression‐free survival (PFS) and other endpoints (treatment response, disease progression, and adverse events) were evaluated. PFS was estimated using the Kaplan‐Meier method. The median treatment duration was 4 months (range, 1‐15). The median PFS for the overall population was 4.2 months (95% CI 2.7‐5.2), while those for patients with wild‐type BRCA1/2 and BRCA1/2 mutations were 3.1 months (95% CI 1.3‐4.6) and 5.3 months (95% CI 2.8‐7.1), respectively. The median PFS tended to be longer for patients on maintenance therapy (between 9.0 months [95% CI 1.4‐17.5] and 10.0 months [95% CI 2.5‐18.1]) than for those on active therapy (between 3.1 months [95% CI 2.1‐3.8] and 3.0 months [95% CI 1.4‐4.5]). Most patients (87.0%) experienced low‐grade adverse events; the most common of which were fatigue (49.0%) and nausea (35.0%).

**Conclusion:**

Our findings demonstrate clinical benefit of olaparib to mainland Chinese patients with high‐grade SOC, particularly for patients with BRCA mutations and who require maintenance therapy.

## 1 INTRODUCTION

Ovarian cancer is one of the most common malignancies of the female reproductive system and the leading cause of death from gynecological tumors.^1^ In China, it was estimated that 52 100 new cases and 22 500 deaths from ovarian cancer would occur in 2015.^2^ High‐grade serous ovarian cancer is the most common type of ovarian cancer. It is associated with aggressive disease progression and poor prognosis, accounting for up to 80% of ovarian cancer deaths.^3^ Although survival rates of ovarian cancer have improved significantly in the last decade due to advancements in surgical techniques and the induction of new chemotherapeutic drugs,^4^, ^5^ disease recurrence and treatment resistance often occur.

About 22% to 26% of patients with serous ovarian cancer present with germline or somatic BRCA1/2 mutations.^6^, ^7^, ^8^, ^9^ BRCA1 and BRCA2 are tumor suppressor genes that regulate the cell cycle and repair DNA double‐strand breaks via homologous recombination. When either BRCA1 or BRCA2 is defective, double‐strand breaks are repaired through alternative mechanisms such as single‐strand repair, which are mediated by poly (ADP‐ribose) polymerase (PARPs).^10^, ^11^, ^12^, ^13^ In BRCA‐mutated cells, inhibition of PARP activity leads to cell death as DNA damages cannot be repaired.^14^ PARP‐inhibitors exploit the concept of “synthetic lethality” between BRCA and PARP to cause tumor cell death.^15^, ^16^ One such example is olaparib, a potent PARP inhibitor that has been shown to have a selective cytotoxic effect on BRCA‐mutant tumor cells in in vitro,^15^, ^16^ in vivo,^17^ and clinical studies.^18^

Olaparib was approved in 2014 for the treatment of patients with deleterious germline BRCA‐mutated advanced ovarian cancer who have received three or more lines of chemotherapy in the United States,^19^ and for maintenance treatment of patients with platinum‐sensitive, BRCA‐mutated (germline and/or somatic), high‐grade serous ovarian cancer who are in complete or partial response to platinum‐based chemotherapy in Europe.^20^, ^21^ More recently, in 2018, olaparib was approved as maintenance treatment for women with platinum‐sensitive relapsed ovarian cancer regardless of BRCA mutation status in Japan^22^ and Europe.^23^ The results of three key prospective, randomized, double‐blind studies (Study 19,^24^ Study 42,^25^ and SOLO‐2^26^) supported these approvals. Both Study 19 and SOLO‐2 reported a significant increase in median progression‐free survival (PFS) in BRCA‐mutated patients treated with olaparib for maintenance therapy with respect to placebo (Study 19: 11.2 months vs 4.3 months^27^; SOLO‐2: 19.1 months vs 5.5 months^26^; both *P* < 0.001), while Study 42 demonstrated a 31.1% response rate and prolonged stable disease in 40.0% of patients with ovarian cancer treated with olaparib.^25^

Olaparib is currently available for use in Macau but not in mainland China. To our knowledge, the use of olaparib in mainland Chinese patients with serous ovarian cancer has not been evaluated. Thus, this study aimed to evaluate the real‐world effectiveness and safety profile of olaparib in patients from mainland China, where olaparib is unavailable.

## 2 MATERIALS AND METHODS

### 2.1 Study design

This single center, observational study investigated patients treated with olaparib between 1 December 2015 and 1 September 2017, who had pathologically confirmed advanced serous ovarian cancer at the Kiang Wu Hospital in Macau. The study protocol and other relevant study‐related documents were reviewed and approved by the Institutional Review Board of the hospital. The study was conducted in compliance with the principles of Declaration of Helsinki, the International Conference on Harmonization Harmonized Tripartite Guideline for Good Clinical Practice, and the applicable local laws and regulations. All patients provided written informed consent prior to study enrollment.

### 2.2 Study population

Inclusion criteria included aged ≥18 years, pathological diagnosis of serous ovarian cancer, undergone surgery and received platinum‐containing chemotherapy, undergone BRCA gene testing, received at least one course of olaparib treatment, and at least one measurable or evaluable lesion or elevated cancer antigen 125 (CA 125) levels, defined as levels above the upper limit of normal (ie, 35 U/mL). Key exclusion criteria included diagnosis of nonepithelial ovarian cancer.

### 2.3 Treatment and follow‐up

Patients received oral olaparib capsules 300 to 400 mg twice daily until disease progression, with each course of treatment lasting 28 days. Patients were treated with a standard dose of 400 mg; 300 mg was prescribed for patients who were unable to tolerate the standard dose. In the event of toxicity, the dose may be adjusted to 250 to 400 mg twice daily, based on patient's tolerability, or discontinued at the investigator's discretion. Patients were followed up monthly until disease progression via regular check‐up by physicians and CT/MRI scans in mainland China, and via telephone as most patients were nonresident in Macau. The last follow‐up date was on 30 May 2018.

### 2.4 Efficacy and safety assessments

The primary efficacy endpoint was PFS. Other efficacy endpoints included treatment response (complete or partial response) and disease progression (stable or progressive disease). Treatment response and disease progression were based on the Response Evaluation Criteria in Solid Tumors (RECIST 1.1).^28^, ^29^ Complete response was defined as the disappearance of all target lesions and reduction of any pathological lymph nodes to <10 mm in short axis; partial response was defined as a minimum of 30% reduction in the sum of diameters of target lesions, relative to the baseline sum diameters. Progressive disease was defined as a minimum of 20% increase in the sum of diameters of target lesions, relative to the smallest sum on study; the sum must also have had an absolute increase of at least 5 mm.^28^ Stable disease was defined as neither sufficient shrinkage (relative to baseline) to qualify for partial or complete response, nor sufficient increase in the sum of diameters (relative to the smallest sum at baseline or on study, whichever is smallest) to qualify for progressive disease.^29^

Treatment response and disease progression were evaluated based on CT/MRI imaging, and the serum marker CA 125 (Gynecologic Cancer InterGroup criteria^30^). CA 125 levels were assessed monthly in a separate institute in mainland China. Safety endpoints included the incidence of adverse events of any grade and of grades III to IV. Patient‐reported adverse events were graded according to the National Cancer Institute Common Terminology Criteria for Adverse Events version 4.0.

### 2.5 Statistical analysis

Demographic variables, baseline disease characteristics, and adverse events were summarized by descriptive statistics. PFS of the overall patient population, according to BRCA1/2 mutation status and according to treatment purpose, were evaluated. The Kaplan‐Meier method was used to estimate PFS, and the log‐rank test was used for differential analysis. Statistical analyses were performed using SPSS version 20.0 software package (SPSS Inc., Chicago, IL, USA). For all analyses, *P* < 0.05 was considered to be statistically significant.

## 3 RESULTS

### 3.1 Patient characteristics

A total of 65 patients were enrolled into the study. Baseline characteristics of these patients are shown in Table 1. Over half of the patients (58.5%) were diagnosed with high‐grade serous ovarian cancer, and 80.0% of patients expressed germline or somatic BRCA mutant gene. The median duration of treatment was 4 months (range, 1‐15), while the median time to follow‐up was 21 months (range, 1‐24). The majority of patients (78.5%) received olaparib as an active therapy for tumor recurrence after at least three lines of chemotherapy.

**Table 1:** Patient demographics and disease characteristics

	All Patients (n = 65)
Age in years, median (range)	53 (24‐73)
Serous ovarian carcinoma grade	
High grade	38 (58.5)
Low/medium grade	12 (18.5)
Unknown	15 (23.1)
BRCA1/2 mutation status	
BRCA mutant^a^	52 (80.0)
BRCA wild‐type	5 (7.7)
Unknown	8 (12.3)
Treatment purpose	
Maintenance therapy	
After 2 lines of chemotherapy	7 (10.8)
After >3 lines of chemotherapy	7 (10.8)
Active therapy for recurrent disease	
After 3 lines of chemotherapy	18 (27.7)
After >4 lines of chemotherapy	33 (50.8)
Platinum response	
Platinum‐sensitive recurrence	38 (58.5)
Platinum‐resistant or refractory recurrence	13 (20.0)
Not available	14 (21.5)
ECOG performance status	
0‐1	36 (55.4)
≥2	29 (44.6)

Values are presented as number (%) unless otherwise stated.

Percentages may not sum to exactly 100 due to rounding.

a Germline or somatic BRCA mutant gene.

### 3.2 Efficacy

**Figure 1:**
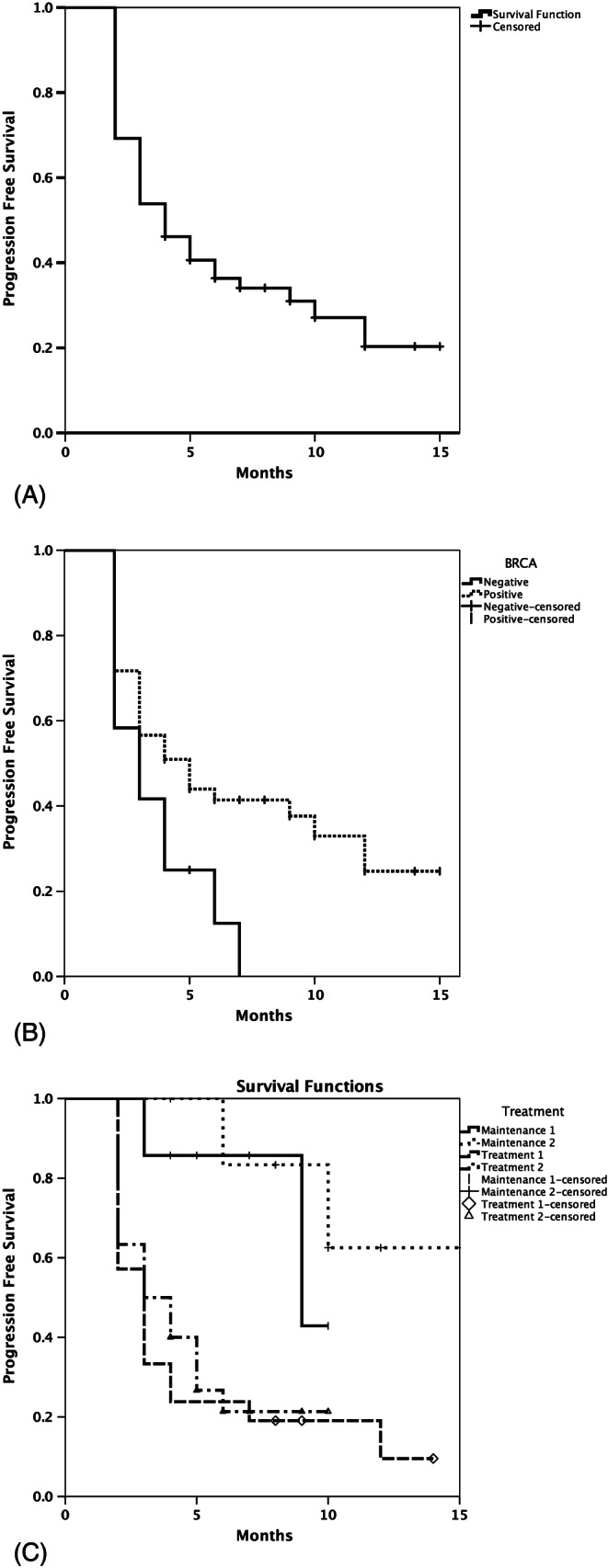
Progression free survival (PFS) curves of (A) overall patient population, (B) according to BRCA1/2 mutation status, and (C) according to treatment purpose

The results for PFS are presented in Figure 1. The median PFS for the overall patient population was 4.2 months (95% CI 2.7‐5.2; Figure 1A). The median PFS for patients who tested positive for BRCA1/2 mutation (5.3 months [95% CI 2.8‐7.1]) tended to be longer than that for patients with wild‐type BRCA1/2 (3.1 months [95% CI 1.3‐4.6]; Figure 1B). Figure 1C shows the PFS for patients who received olaparib as maintenance or active therapy. Overall, the median PFS for patients who received olaparib for maintenance therapy after two (9.0 months [95% CI 1.4‐17.5]) and more than three lines of chemotherapy (10.0 months [95% CI 2.5‐18.1]) tended to be longer than that for patients who received olaparib for active therapy after three (3.1 months [95% CI 2.1‐3.8]) and more than four lines of chemotherapy (3.0 months [95% CI 1.4‐4.5]).

A summary of the patients' response to olaparib treatment is provided in Table 2. Most patients (41.5%) had progressive disease. The remaining patients had partial response (21.5%) or stable disease (36.9%). At the end of follow‐up, three patients (5%) were still on treatment at end of follow‐up. They maintained response or disease control under Olaparib therapy. CA125 response data was not shown because we noticed the variable limits between different hospital where these patients were followed up.

**Table 2:** Response and disease progression with olaparib treatment

	All Patients (N = 65)
Treatment response^a^	
Complete response	0 (0.0%)
Partial response	14 (21.5%)
Disease progression^a^	
Stable disease	24 (36.9%)
Progressive disease	27 (41.5%)

Values are presented as number (%).

Percentages may not sum to exactly 100 due to rounding.

a Assessed in accordance with the Response Evaluation Criteria in Solid Tumors 1.1.

### 3.3 Safety

A summary of adverse events is provided in Table 3. Of the 65 patients, two‐thirds (66%) experienced adverse events, and 13% experienced grade III to IV adverse events. The most common adverse events were fatigue (49%), gastrointestinal disturbances (nausea: 35%; vomiting: 14%), and leukopenia (20%). Three patients (5%) had dose reductions due to adverse events. Three patients (5%) discontinued olaparib treatment due to pain and/or disease progression.

**Table 3:** Summary of adverse events

	Any Grade	Grade III‐IV
Adverse events	43 (66%)	9 (13%)
Drug‐related adverse events	36 (55%)	5 (7%)
Most common adverse events^a^		
Leukopenia	13 (20%)	4 (6%)
Thrombocytopenia	7 (10%)	2 (3%)
Nausea	23 (35%)	4 (6%)
Vomiting	9 (14%)	2 (3%)
Fatigue	32 (49%)	3 (4%)
Pain	5 (7%)	3 (4%)
Rashes	4 (6%)	0 (0%)

Values are presented as number (%).

a Defined as an adverse event that occurred in >5% of patients.

## 4 DISCUSSION

The present study sought to investigate the real‐world effectiveness and safety of olaparib in Chinese patients with advanced serous ovarian cancer previously treated in mainland China, where olaparib is unavailable. Our findings showed that the median PFS for patients who received olaparib for maintenance therapy tended to be longer than that for patients who received olaparib for active therapy. Patients with BRCA1/2 mutations also tended to have longer median PFS than those with BRCA1/2wild‐type. Although adverse events occurred in two‐thirds (66%) of patients, the majority of patients (87%) experienced low grade adverse events (grades I‐II). The most common adverse events were fatigue and gastrointestinal disturbances (49% for both).

The median PFS with olaparib as a maintenance therapy after at least two lines of chemotherapy in the present study (9.0 months) is consistent with published data from studies of maintenance treatment in a similar patient population (8.4 months).^24^ Median PFS for patients who received olaparib for active therapy after at least three lines of chemotherapy tended to be shorter than that for maintenance therapy, as expected. However, the median PFS of olaparib active therapy in our study (approximately 3 months) was less than half of those reported in previous clinical trials (6.5 to 8.5 months^25^, ^31^). The proportion of patients who had a complete or partial response (21.5%) and who had stable disease (36.9%) observed in our study were also lower than those reported in previous clinical trials (Study 42: response rate: 31.1%, patients with stable disease: 40.0%^25^; Kaye et al: response rate: 31.0%, patients with stable disease: 59.0%^31^). Notably, the majority of patients in our study had high grade serous ovarian cancer (58.5%), were heavily pretreated (>50% had four or more lines of prior chemotherapy), and were in poor health at baseline (44.6% had ECOG status ≥2), which may explain the shorter PFS and lower response rates observed. In addition, the duration of treatment (4 months) was much shorter than that in published clinical trials where olaparib was continued until disease progression. The short duration of treatment in the present study suggests poor adherence, perhaps due to high cost of medication (nonreimbursable for mainland China patients) as well as cross‐border treatment, and suboptimal remote follow‐up. Access to local treatment and follow‐up may help to improve treatment prospects and outcomes for patients with serous ovarian cancer in mainland China.

In the pivotal study by Ledermann et al,^24^ a retrospective subgroup analysis found that patients with BRCA1/2 mutations derived the greatest clinical benefit from olaparib (ie, significantly longer PFS compared with those with BRCA1/2wild‐type). In the present study, most patients (80.0%) had BRCA mutations, while the remaining had unknown BRCA mutation status (12.3%) or were BRCA wild‐type (7.7%). As expected, subgroup analyses found that median PFS for BRCA‐mutated patients tended to be longer than that for BRCA wild‐type patients (5.3 months vs 3.1 months). The differences in PFS, however, were not significant, likely due to the small number of BRCA wild‐type patients in this study. At present, the majority of test centers in China use a second‐generationhigh‐throughput sequencing technique to detect BRCA mutations and to classify mutations into one of the following categories: pathogenic mutation, suspected pathogenic mutation, mutation of unknown clinical significance, suspected benign mutation, and benign mutation.^32^ This approach is cost‐efficient but does not detect structural variations and variations in gene regulatory regions of the BRCA gene.^33^ Furthermore, due to the lack of genetic experts in the country, interpretation of BRCA test results can be challenging.^34^ These aforementioned challenges are evident in our study, where approximately one in 10 patients had BRCA mutations that were classified to be of unknown clinical significance. Establishing a test standard for determining BRCA mutation status would be vital for guiding management and for informing clinical treatment decisions for patients with ovarian cancer in China.

The safety profile of olaparib in our study was consistent with that reported in previous studies.^24^, ^25^, ^31^ Similar to what others have found, the most common adverse events reported were fatigue, nausea, and vomiting, and the majority of such events were of mild to moderate severity (grade I‐II). A small proportion of patients (13%) in our study reported grade III to IV adverse events. Guidelines recommend a stepwise dose reduction of olaparib capsules to manage adverse events, starting with 200 mg twice daily to 100 mg twice daily.^35^, ^36^ Kaufman et al adopted this dose reduction regimen for managing olaparib‐related toxicities.^25^ In this study, we observed improvements in symptoms of severe fatigue and gastrointestinal disturbances after olaparib dose reduction from 400 to 300 mg. We also noted that a smaller proportion of patients (5.0%) had to reduce olaparib dose due to adverse events, compared with that in Study 19 (22.8%) and Study 42 (40.3%). Further studies are required to confirm the optimal dosing regimen for olaparib in the patients who experience treatment‐related adverse events, and who have comorbidities and poor performance status. More importantly, physicians need to recognize that responses to treatment vary between individuals, and that personalized dosing regimens may be necessary for optimal treatment outcomes.

Interpretations made here may be limited due to the short median duration of treatment in our study (4 months) compared with those in previous studies (8.7 months^24^ and 19.4 months^26^). Furthermore, the heterogeneity of our patient population and short time to follow‐up (21 months) may have underestimated treatment effects and limited observations relating to long‐term safety outcomes, respectively. Nevertheless, our study is the first to investigate the real‐worldeffectiveness and safety profile of olaparib in Chinese patients with serous ovarian cancer and provides important insights into the use of olaparib in a largely different ethnic patient population compared with those studied in previous prospective trials. Ongoing trials are exploring olaparib for first‐line, maintenance treatment of serous ovarian cancer (SOLO‐1 trial; NCT01844986), or in combination with other chemotherapeutic agents such as bevacizumab (PAOLA‐1 trial; NCT02477644).^37^ Future studies may explore the possibility of olaparib for first‐line, active treatment of serous ovarian cancer, or in combination with other targeted drugs.

In conclusion, our analysis of real‐world treatment outcomes of olaparib treatment in mainland Chinese patients with high‐grade serous ovarian cancer demonstrated clinical benefit, especially for BRCA‐mutated patients and patients requiring maintenance therapy. Greater access to olaparib may help to improve treatment prospects and outcomes for patients with serous ovarian cancer in mainland China.

## AUTHORS' CONTRIBUTIONS

All authors had full access to the data in the study and take responsibility for the integrity of the data and the accuracy of the data analysis. *Conceptualization*, Y.C.; *Methodology*, Y.C.; *Investigation*, Y.C.; *Formal Analysis*, Y.C.; *Resources*, Y.C., H.H.; *Writing ‐ Original Draft*, Y.C.; *Writing ‐ Review & Editing*, Y.C., H.H.; *Visualization*, Y.C.; *Supervision*, Y.C.; *Funding Acquisition*, Y.C.

## CONFLICT OF INTEREST

The authors declare that they have no competing interests and no relevant relationships to disclose.

## CONSENT FOR PUBLICATION

We have obtained consent from the participants to publish and report individual patient data.

## AVAILABILITY OF DATA AND MATERIAL

The authors confirm that, for approved reasons, some access restrictions apply to the data underlying the findings. The relevant data underlying this paper contain clinical patient information. The data are available to all interested parties upon written request to the Kiang Wu Hospital.
